# Advancements in Artificial Intelligence (AI) for Cancer Genomics and Genetics

**DOI:** 10.3390/biomedicines14020274

**Published:** 2026-01-26

**Authors:** Roberto Piergentili

**Affiliations:** Istituto di Biologia e Patologia Molecolari (IBPM) del Consiglio Nazionale delle Ricerche (CNR), at Dipartimento di Biologia e Biotecnologie, Università Sapienza di Roma, Piazzale Aldo Moro 5, 00185 Rome, Italy; roberto.piergentili@cnr.it

Advances in omics disciplines—particularly those directly involved in gene function and regulation, including genomics, epigenomics, exomics, transcriptomics, and proteomics, as well as fields addressing genomic responses to pharmacological interventions such as pharmacogenomics and metabolomics—have enabled an increasingly comprehensive characterization of gene activity in health and disease. In parallel, improvements in data acquisition technologies have generated vast and highly heterogeneous datasets that require advanced computational approaches for their systematic management, integration, analysis, and interpretation. The development of robust, data-driven models is therefore essential to elucidate disease mechanisms at the molecular level and to establish the foundations of personalized medicine.

Cancer research, and particularly the study of neoplastic transformation, has significantly benefited from these technological and methodological advances; nevertheless, a fully integrated and dynamic understanding of cancer initiation and progression remains incomplete. Cancer is a complex, multistep disease whose onset and evolution are determined by the interplay of genetic background, lifestyle, environmental factors, and their intricate interactions. Consequently, cancer characterization can no longer be confined to cytological or histopathological evaluation and the assessment of a limited number of biomarkers. Instead, a systems-level, integrative approach is required, combining high-throughput data generation with contextualized, multi-dimensional data analysis.

The effective exploitation of such complex datasets necessitates broad expertise and strong interdisciplinary collaboration, as the scale and heterogeneity of the data exceed the capabilities of individual laboratories. In this context, Artificial Intelligence (AI), including machine learning and deep learning methodologies, has emerged as a powerful and increasingly necessary tool for extracting meaningful patterns from large datasets. These approaches are enabling improved disease modeling and are increasingly supporting predictive analysis, decision support, drug discovery, assessment of treatment response, and the development of more accurate diagnostic, prognostic, and therapeutic strategies.

Here, we are proud to present our guest-edited Special Issue, “Advancements in Artificial Intelligence (AI) for Cancer Genomics and Genetics”, presenting the most recent reports on the use of Artificial Intelligence to investigate the etiopathogenesis of cancer. In this issue, readers will find six contributions, which may be accessed at the following link: https://www.mdpi.com/journal/biomedicines/special_issues/WI9U880UY5.

Aznar-Gimeno and collaborators developed GastricAITool, a clinical decision support system for the diagnosis and prognosis of gastric cancer (GC) [1]. Utilizing algorithms such as XGBoost and Random Survival Forest, the model integrates clinical, demographic, and genetic data (SNPs), demonstrating that the inclusion of genetic information significantly enhances the system’s discriminatory capacity. The application of Explainable AI (XAI) techniques ensures transparency in the model’s decision-making processes. Future research aims to implement self-learning algorithms (SLAs) for continuous optimization with new data, as well as the integration of medical imaging and immunotherapy data to further refine diagnostic precision.

Truntzer and collaborators generated a deep learning approach based on whole-slide images (WSIs) to predict the outcomes of patients with pancreatic ductal adenocarcinoma (PDAC) following surgery [2]. This multi-instance learning model identified two patient groups with distinct prognoses, associating the poor-prognosis group with a squamous phenotype. The study emphasizes the necessity for validation in prospective series of patients treated with modern regimens, such as FOLFIRINOX, to confirm the model’s actual predictive performance.

Spirina Menand and collaborators explored the N-MTLR-Rank discrete survival model applied to ovarian cancer transcriptomic data [3]. Using PatternAttribution methodology, they identified six molecular pathways (Hallmark pathways), including MTORC1 signaling and E2F targets, capable of stratifying patients into high- and low-risk groups. While current models face challenges in generalization, these pathways serve as powerful hypothesis generators for future clinical and in vitro studies about molecular pathogenesis.

Yang and collaborators developed AI-HOPE-RTK-RAS, a conversational AI system based on large language models (LLMs) for the integrated analysis of RTK-RAS pathway alterations in colorectal cancer (CRC) [4]. The tool revealed a lower prevalence of such alterations in early-onset CRC (EOCRC) patients and identified non-canonical mutations (CBL, NF1) specific to certain ancestries. Future objectives include extending the platform’s capabilities to other oncogenic pathways, integrate electronic health record (EHR) data, and implement functions for AI-assisted clinical trial matching.

Lin and collaborators describe the ARIMA-CNN framework to integrate the temporal dynamics of CCL5 gene expression with immune signatures in hepatocellular carcinoma (HCC) [5]. The model demonstrated that immune clusters (CD8+ T cells and Th1) synergistically influence survival, outperforming traditional single-gene analyses and paving the way for precision immunotherapy strategies based on the dynamic modeling of the tumor immune microenvironment.

Finally, Slalmi and collaborators present a systematic review aimed at evaluating the use of AI in the SELEX process for designing aptamer panels intended for the detection of urinary biomarkers in prostate cancer [6]. AI integration reduced enrichment cycles by 40–55% and optimized aptamer affinity for targets such as PCA3 and extracellular vesicles. Priorities for future perspectives include the standardization of urinary extracellular vesicle (uEV) processing and multicenter external validation to facilitate the clinical adoption of these biosensors.

The collection of studies presented in this edition highlights how the integration of clinical, genomic, and transcriptomic data mediated by Artificial Intelligence is redefining our understanding of cancer. The importance of this research lies in its capacity to transform complex data into useful clinical insights, allowing for more refined patient stratification and the identification of previously obscure resistance mechanisms. These studies not only provide more precise diagnostic and prognostic tools but also establish a new paradigm for precision medicine. The transition from static models to dynamic and conversational systems promises to allow access to multi-omic analysis, accelerating biomarker discovery and the optimization of personalized treatments, thereby marking a fundamental step in the global fight against neoplastic diseases.

We are grateful to all authors who submitted their work and supported this collection, and to the Biomedicines staff, whose help was invaluable for the success of this editorial project. All articles are open access to readers and distributed under the terms and conditions of the Creative Commons Attribution (CC BY) license 4.0 (https://creativecommons.org/licenses/by/4.0/).
